# Buffalo Medical College

**Published:** 1888-04

**Authors:** 


### Editorial.

---

### *BUFFALO MEDICAL COLLEGE.*

The annual commencement occurred on the 28th of February. The alumni of the college assembled in goodly numbers, and were welcomed by Dr. Mann on behalf of the Faculty. After a graceful tribute to the memory of Dr. Rochester, Prof.

Mann alluded in glowing terms to the present incumbent of the chair, as one who had proved himself a worthy successor to Flint and Rochester. We are gratified, indeed, to note that our predictions as to the position which Dr. Stockton would take among his colleagues in the Buffalo Medical College, have been so abundantly fulfilled. His innate ability and the advantages which he had derived from his earnest efforts, in conjunction with his late confreres of Niagara University, to establish a more thorough system of medical education, convinced us that he could easily take the first place in the old college, not only with credit to himself, but also to the great advantage of the institution. Without intending to throw too great a responsibility upon Dr. Stockton, we cannot but recall with pleasure our prediction, that his appointment would lead to the gradual introduction of more rigid examinations and a more faithful performance of the great responsibilities to the community and to the profession, which devolve upon the professors of our medical colleges. Last year, we had occasion to note the fact that every candidate for graduation had triumphantly passed all examinations. This year, it is announced that fifteen per cent. of the candidates were rejected, and that in the ensuing year the course is to be lengthened. This is progress indeed, and we predict that it will please every alumnus of the college. We regret, however, that sufficient courage has not yet been instilled to lead to the elevation of this school from the ranks of the "two-year class, requiring no matriculation examination," whose diplomas will not be recognized in many of the states. The alumni have the right to demand that the college shall keep abreast of other reputable institutions, and it may serve to stimulate the authorities of the college, to be informed of the rapid advances being made by sister colleges. The last report of the State Board of Health of Illinois shows that there are now one hundred and fourteen medical schools which exact an educational preparation of intending matriculates, as against forty-five formerly. Forty-three colleges now require at least three courses of lectures, as against twenty-two formerly, and fifty-seven provide for a three or four years' graded course.

Sixty-three schools have now terms of six months or more. The following portion of Dr. Mann's address to the alumni would indicate that the sentiments of the faculty are all in favor of the higher medical education, but that there is a fear that in the adoption of a proper standard they would place the college too far in advance of the alumni. We quote:

"In our endeavor to raise the standard of medical education in our college, we ask your assistance, which you can give by urging your students to take a three-years' course, and by sending only those who are already well prepared in the preliminary education. Until there is some general law on the subject, or some community of action among the medical schools of the state, all attempts at raising the standard must be by slow degrees. We can go no faster than you will follow. It is with the practicing physicians of the state that the solution of the question lies. We, the faculty, are willing to go as fast and as far as we can get your support."

We earnestly hope that, at the next meeting of the alumni, a resolution will be introduced requesting the authorities of the college to make a three-years' course obligatory. We are confident that such a resolution would meet with almost unanimous favor, and, with this statement of the earnest "endeavor to raise the standard of medical education in our college," on the part of members of the faculty, it would appear to be only necessary for the alumni to assure them of their hearty support and sympathy in the plan. Undoubtedly, it would, for a time, lessen the fees which the professors would receive, but we would not for a moment believe that the loss of a paltry hundred or two of dollars would influence the distinguished members of the faculty against that which would be for the elevation of the college and the advancement of medical education.

The following gentlemen were elected as officers of the Alumni Association for the ensuing year:

*President*—D. W. Harrington; *First Vice-President*—E. L. Shurley, Detroit; *Second Vice-President*—J. H. Pryor; *Third Vice-President*—T. D. Strong, Westfield; *Fourth Vice-President*—E. N. Moore, Jr.,

Rochester; *Fifth Vice-President*—Peter Enweis, Ovid; *Permanent Secretary*—J. J. Walsh; *Treasurer*—E. C. W. O'Brien; *Recording Secretary*—J. W. Matzinger; *Trustee for five years*—F. E. L. Brecht; *Executive*—F. P. Vandenburgh, H. H. Bingham, W. C. Callanan.

The commencement exercises were held at Music Hall; the pen of the reporter of one of our great dailies is here so facile that we give place to him: "It was a scene of unaccustomed brilliancy. Upon the stage, set as a library scene, were grouped the faculty of the College, the curators, and the speakers of the the evening; the three front rows of chairs were occupied by members of the classes in medicine and pharmacy; Wahle's orchestra was stationed inside of the railing, ready to intersperse the eloquence of the orators with sweet music; a large collection of bouquets and floral pieces was displayed beside the canvas-covered steps leading to the stage, ready for distribution as soon as the diplomas had been awarded; the boxes were filled with the families of leading physicians, while stretched away to the very doorways on the main floor was a sea of faces, all lighted with pride and expectancy. After an orchestral selection, the exercises were opened with an eloquent prayer by Bishop Hurst."

The address by the Hon. Theodore Bacon, of Rochester, was worthy of his high reputation as an able and scholarly man. The following gentlemen then received their diplomas:

Edward L. Wood, Eden, N. Y.; Henry George Bentz, Buffalo, N. Y.; Arthur Lincoln Benedict, A. B., Buffalo, N. Y.; Charles H. Farrington, Holland, N. Y.; Ira C. Brown, Buffalo, N. Y.; T. Oliver Tait, Rochester, N. Y.; J. Frederick Haller, Jamestown, N. Y.; Grant H. Simonds, Shelby, N. Y.; Charles E. Sharp, Port Allegany, Pa.; Frank L. Cooley, Hannibal Center, N. Y.; Ransford C. Tabor, Tonawanda, N. Y.; Fred. A. Wicker, Moscow, N. Y.; W. Henry Woodbury, A. B., Stamford, Conn., Horace F. Smith, Allanburg, Canada; Charles D. McCarthy, E. Bloomfield, N. Y.; Marcenus Mason, E. Bloomfield, N. Y.; Oscar L. Harries, Buffalo, N. Y.; Alfred W. Smallman, Ellicottville, N. Y.; Charles B. B. Tweedale, St. Thomas, Canada; Charles S. Jones, Middlesex, N. Y.; Thomas Purcell, Lundy's Lane, Pa.; Geo. W. Gillette, Ph. B.,

Buffalo, N. Y.; Amos F. Blanchard, Jamestown, N. Y.; Edward Beck, Owego, N. Y.; Wm. E. McDuffee, Otto, N. Y.; Charles George G. Le Vesconte, Kingscave, N. F.; William McGregor Haynes, Beamsville, Canada; Charles Maynard Smith, Westfield, Mass.; Ellen R. Spragge, Guelph, Canada; Thomas H. Davidson, Buffalo, N. Y.; Horace J. Mann, Brockport, N. Y.; Frank B. Storer, E. Kendall, N. Y.; Herbert L. Smith, S. Rutland, N. Y.; Ava Melissa Carroll, Grant Station, N. Y.; Robert T. French, Jr., A. M., Rochester, N. Y.; Joseph Benjamin Finucan, Mendon, N. Y.; Hiram L. Knapp, Windham, Pa.; Elizabeth Fear, Dansville, N. Y.; Richard Henry Satterlee, Rochester, N. Y.; Charles H. Andrews, Bergen, N. Y.; Clark Edwin Ernest, Lockport, N. Y.; Edward Hadley Tweedy, Buffalo, N. Y.; Peter Cortelyou Cornell, Buffalo, N. Y.; Selina P. Colgrove, Salamanca, N. Y.

### ALUMNI ASSOCIATION PRIZE.

An annual prize of two hundred dollars is offered by the Alumni Association of the Medical Department of the University of Buffalo for an original essay on any medical or surgical subject. The prize is open for competition to the Alumni of the Medical Department only.

Each essay must be signed by a motto, and accompanied by a sealed envelope containing the author's name and address, and bearing on the outside the motto with which his essay is signed.

The author and the subject of the successful essay will be announced, and the prize awarded, at the regular annual meeting of the Alumni Association.

The Committee reserves the right of not awarding the prize at all, if no essay of sufficient value is presented.

All essays should be sent to the Secretary of the Committee before January 1, 1889.

JOHN H. PRYOR, M. D., *Chairman*,
WM. H. THORNTON, M. D.,
DE LANCEY ROCHESTER, M. D., *Secretary*,
216 Franklin street, Buffalo, N. Y.

*Committee.*

